# Facial emotion processing in major depression: a systematic review of neuroimaging findings

**DOI:** 10.1186/2045-5380-1-10

**Published:** 2011-11-07

**Authors:** Anja Stuhrmann, Thomas Suslow, Udo Dannlowski

**Affiliations:** 1 University of Münster, Department of Psychiatry, Albert-Schweitzer-Campus 1, Building, A9, 48149 Münster, Germany; 2 University of Leipzig, Department of Psychosomatic Medicine and Psychotherapy, Semmelweisstraße 10, 04103 Leipzig, Germany

**Keywords:** Facial emotion processing, fMRI, neuroimaging, depression, emotion, amygdala, anterior cingulate, orbitofrontal cortex, functional connectivity

## Abstract

**Background:**

Cognitive models of depression suggest that major depression is characterized by biased facial emotion processing, making facial stimuli particularly valuable for neuroimaging research on the neurobiological correlates of depression. The present review provides an overview of functional neuroimaging studies on abnormal facial emotion processing in major depression. Our main objective was to describe neurobiological differences between depressed patients with major depressive disorder (MDD) and healthy controls (HCs) regarding brain responsiveness to facial expressions and, furthermore, to delineate altered neural activation patterns associated with mood-congruent processing bias and to integrate these data with recent functional connectivity results. We further discuss methodological aspects potentially explaining the heterogeneity of results.

**Methods:**

A Medline search was performed up to August 2011 in order to identify studies on emotional face processing in acutely depressed patients compared with HCs. A total of 25 studies using functional magnetic resonance imaging were reviewed.

**Results:**

The analysis of neural activation data showed abnormalities in MDD patients in a common face processing network, pointing to mood-congruent processing bias (hyperactivation to negative and hypoactivation to positive stimuli) particularly in the amygdala, insula, parahippocampal gyrus, fusiform face area, and putamen. Furthermore, abnormal activation patterns were repeatedly found in parts of the cingulate gyrus and the orbitofrontal cortex, which are extended by investigations implementing functional connectivity analysis. However, despite several converging findings, some inconsistencies are observed, particularly in prefrontal areas, probably caused by heterogeneities in paradigms and patient samples.

**Conclusions:**

Further studies in remitted patients and high-risk samples are required to discern whether the described abnormalities represent state or trait characteristics of depression.

## Background

Major depression ranks among the most debilitating diseases worldwide and is estimated to produce the second largest disease burden by the year 2020 [1]. Despite an increasing amount of empirical studies investigating abnormalities in affective processing in unipolar depression, understanding the neurobiological underpinnings is still a major research goal and is essential for novel treatment developments. In a large body of behavioral studies, depression has been characterized by mood congruent emotion processing biases in different aspects of cognition [2-5]. Apparently, these cognitive biases have been reported to be particularly prominent for emotional faces. Depressed patients seem to be less sensitive in the identification of emotional faces and, in addition, a negative response bias was found: they tend to interpret neutral faces as sad and happy faces as neutral (for review see [6,7]).

**Figure 1:**
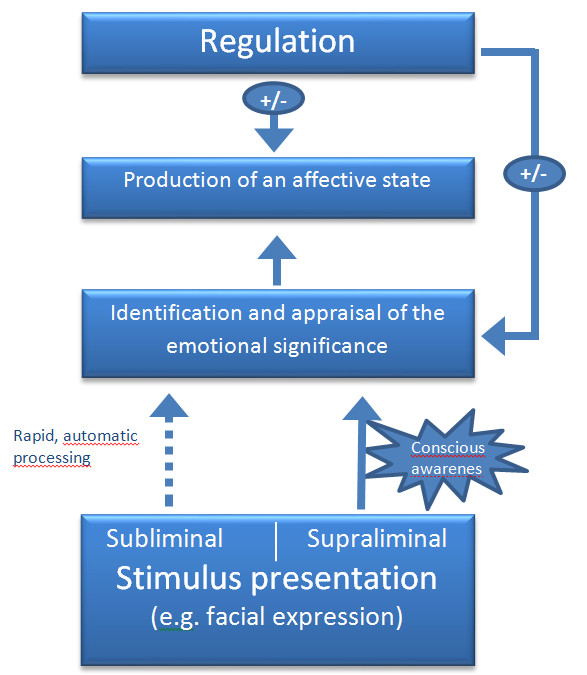
**Emotional perception and processing stages**. After stimulus presentation (subliminal or supraliminal) the central emotion perception and processing stages are: (1) the identification and appraisal of stimulus significance, taking place with or without conscious awareness; (2) the generation of an affective state, expression of emotion and behavioral response; and (3) up or down regulation (circles with positive/negative signs) of the affective state and identification process. Modified from Phillips *et al*. [15].

While negative faces seem to be processed more rapidly and deeply, processing of positive facial expressions appears to be impaired [8-10]. Furthermore, behavioral biases towards sad faces seem to persist even after recovery from depression [11], increasing the risk for future depressive episodes [12]. Interestingly, rapid, automatic stages of emotion processing are also affected in depression, as suggested by studies employing subliminal presentation conditions [13,14]. Figure 1 presents the main emotion processing stages as supposed by Phillips *et al*. [15], extended about separate pathways for stimulus presentation with or without conscious awareness.

Faces are a very important component of daily human visual communication. Since the processing of facial expressions is a fundamental step in social functioning, guiding adequate social interaction [16], biased processing of emotional faces in depression could be a strong determinant of the frequently observed interpersonal problems, including social withdrawal, feelings of interpersonal rejection and restriction of non-verbal expressiveness [17].

Brain imaging techniques, such as functional magnetic resonance imaging (fMRI), have already made substantial contributions to the understanding of how faces and facial expressions are processed in humans [18-21]. According to neurobiological models of emotional face processing, successful encoding of emotional expressions depends on multiple interactions between complimentary systems: a neural core system for the visual analysis of faces consists of the bilateral inferior occipital gyrus, the lateral fusiform gyrus and the superior temporal sulcus. Changeable and invariant aspects of the face representation have distinct representations in this system. A second, extended system supports the processing of facial information such as meaning and significance. It is composed of additional brain areas generally involved in representing and producing emotions. Major components include the amygdala, insula, orbitofrontal areas and somatosensory cortex [22]. Notably, most if not all of these areas have already been implicated in the etiology of major depression (see [23-25] for reviews). Thus, presenting facial emotional stimuli is a valid and reliable approach in order to activate brain areas crucial for emotion processing in general and crucial for the pathophysiology of depression specifically [18]. Unsurprisingly, emotional faces have been frequently employed in neuroimaging studies in depressed patients, contributing to the refinement of neurobiological models of depression [24-26]. Put simply, these models postulate increased activity in brain regions essential for emotional identification and production (that is, amygdala, orbitofrontal cortex (OFC), striatum) and decreased neural activity within regions important for emotion regulation such as the dorsolateral prefrontal cortex (DLPFC) and anterior cingulate cortex (ACC).

However, currently available data on emotional face processing in depression are far from being consistent. The heterogeneity of study samples (for example, state of illness, medication status and so on), imaging paradigms (for example, implicit or explicit processing paradigms, stimulus material, baseline condition), and analysis strategies (for example, activation or connectivity analyses) is reflected in apparently heterogeneous and partly conflicting findings at first sight. Given the importance of emotional face processing in major depression, the goal of the present review is to provide a comprehensive overview of neuroimaging studies investigating facial emotion processing in acutely depressed patients compared with healthy controls. Particular effort was made to delineate altered neural activation patterns associated with mood-congruent processing bias and to integrate these findings with functional connectivity results.

First, we describe in detail the results of all available fMRI studies comparing facial emotion-related brain activation in patients with major depressive disorder (MDD) and healthy control (HC) subjects. In addition to whole brain and region of interest (ROI) data, recent functional connectivity data will also be considered. Finally, the summarized results will be discussed in the context of current models of depression and their possible role for 'trait' or 'state' aspects of depression.

## Methods

To identify relevant functional neuroimaging studies focusing on emotional face processing in major depression, a database search of journal articles via Medline, Embase and Scopus was conducted from the year 2000 to August 2011. We used combinations of the keywords 'fMRI', 'functional magnetic resonance', 'depression', 'MDD', 'face', 'facial expression', and 'emotion'. All studies were limited to English language publications. We further examined the reference lists of review articles on MDD and all studies identified for inclusion to check for potentially useful studies not identified by computerized literature search.

Studies were included if they: (1) were fMRI studies, (2) statistically compared a group of adult patients with MDD to a group of healthy volunteers (3) utilized facial emotion expressions as stimuli (4) conducted a whole brain analysis, ROI analysis or functional connectivity analysis (5) reported results in acute depression (during current episode). Thus, we did not consider results reported in remitted patients. We did not include fMRI studies simply correlating imaging data with clinical features without any comparison to HCs.

Variables of interest extracted from the studies were differences in neural activations during facial emotion processing in MDD patients compared to HCs. Therefore, we extracted the neuroimaging data of between-group comparisons regarding experimental conditions reflected by 'emotion vs baseline' contrasts (for example, MDD > HC, HC > MDD).

## Results

The literature search yielded a total of 25 studies meeting the inclusion criteria (see Table 1). A total of 20 studies reported between-group results in terms of whole brain and/or ROI data, whereas only 1 study found no differences between MDD patients and the healthy control group at a pretreatment baseline [27]. Functional connectivity data were reported by six studies. One study reported both whole-brain and FC results [28].

**Table 1:** Description of fMRI studies on facial emotion processing, comparing a group of major depressive disorder (MDD) patients to healthy controls (HCs)

Author/year	Reference	Participants	Patient mean age (SD)	Patient (a) mean duration of illness in months; (b) mean episodes	Medication	Emotions	Paradigm and stimulus type	Stimulus duration	Analysis approach
Whole brain and/or ROI data:									

Almeida *et al*. 2010	[62]	15 MDD, 15 HC, (15 BDD), (15 BDDr)	32.74 (9.87)	(a) 13.67 ± 9.87; (b) not reported	Yes	Fear, sad, happy	Facial expression processing paradigm. Ekman faces. Morphed 50% and 100% intensity. Explicit task: label emotion.	2 s	ROI

Frodl *et al*. 2009	[43]	12 MDD, 12 HC	43.3 (11.2)	Not reported	Yes	Sad, angry	Emotion face-matching task. Ekman faces. Explicit task: match emotion. Implicit task: match gender. Control task: match shapes.	5.3 s	Whole brain, ROIs

Frodl *et al*. 2011	[27]	24 MDD, 15 HC	38.9 (10.4)	(a) 56.0 ± 63.4; (b) 1.6 ± 0.7	No	Sad, angry	Emotion face-matching task. Faces from Gur and colleagues. Explicit task: match the emotion. Implicit task: match the gender. Control task: match shapes.	5.3 s	Whole brain

Fu *et al*. 2004; Fu *et al*. 2007	[32,63]	19 MDD, 19 HC	43.2 (8.8)	Not reported	No	Sad, happy	Facial expression processing paradigm. Ekman faces. Morphed to express low, medium and high intensities. Implicit task: indicate the sex of the face.	3 s	Whole brain

Fu *et al*. 2008	[33]	16 MDD, 16 HC	40.0 (9.4)	(a) not reported; (b) 0.63	No	Sad	Facial expression processing paradigm. Ekman faces. Morphed to express low, medium and high intensities. Implicit task: indicate the sex of the face.	3 s	Whole brain

Gotlib *et al*. 2005	[45]	18 MDD, 18 HC	35.2	Not reported	Yes	Sad, happy, neutral	Facial expression processing paradigm. Ekman faces. Implicit task: indicate the sex of the face.	3 s	Whole brain

Keedwell *et al*. 2005	[42]	12 MDD, 12 HC	43 (9.8)	Not reported	Yes	Sad, happy, neutral	Mood provocation paradigm. Individual autobiographical memory prompts played prior to the presentation of mood congruent facial expressions. Ekman faces. Task: oral subjective rating of mood.	2 s	Whole brain

Lawrence *et al*. 2004	[37]	9 MDD, 11 HC, (12 BDD)	41^a ^(11)	(a) 96 ± 60; (b) not reported	Yes	Sad, fear, happy, neutral	Facial expression processing paradigm. Ekman faces. Morphed 50% and 100% intensity. Implicit task: indicate the sex of the face.	2 s	Whole brain, ROIs

Lee *et al*. 2008	[38]	21 MDD, 15 HC	46.8 (9.1)	(a) 14.8 ± 3.3; (b) 1.9 ± 0.8	Yes	Sad, angry, neutral	Face viewing paradigm. Data set of Korean faces. Task: evaluative ratings (arousal, valence).	1.5 s	ROIs

Matthews *et al*. 2008	[28]	15 MDD, 16 HC	24.5 (5.5)	(a) not reported; (b) 4.46	No	Angry, fear, happy	Emotion face-matching task. Emotional faces. Task: match faces.	5 s	ROI

Peluso *et al*. 2009	[35]	14 MDD, 15 HC	37.9 (14)	Not reported	No	Angry, fear	Emotion face-matching task. Ekman faces. Explicit task: match emotion. Implicit task: match faces. Control task: match shapes.	5 s	Whole brain, ROI

Scheuerecker *et al*. 2010	[41]	13 MDD, 15 HC	37.9 (10.1)	(a) 52.3 ± 71.5; (b) 1.45 ± 0.68	No	Sad, angry	Emotion face-matching task. Faces from Gur and colleagues. Explicit task: match the emotion. Implicit task: match the gender. Control task: match shapes.		Whole brain

Sheline *et al*. 2001	[34]	11 MDD, 11 HC	40.3	Not reported	No	Fear, happy, neutral	Subliminal emotion paradigm. Masked Ekman faces. Task: indicate the sex of the face.	Prime: 40 ms; mask: 160 ms	ROI

Surguladze *et al*. 2010	[39]	9 MDD, 9 HC	42.8 (7.2)	(a) 96 ± 61.2; (b) not reported	Yes	Disgust, fear, neutral	Facial expression processing paradigm. Ekman faces. Morphed 50% and 100% intensity. Implicit task: indicate the sex of the face + offline facial affect recognition task.	2 s	Whole brain

Surguladze *et al*. 2005	[31]	16 MDD, 14 HC	42.3 (8.4)	(a) 90 ± 61.2; (b) not reported	Unknown	Sad, happy, neutral	Facial expression processing paradigm. Ekman faces. Morphed 50% and 100% intensity. Implicit task: indicate the sex of the face.	2 s	Whole brain, ROIs

Suslow *et al*. 2010	[30]	30 MDD, 26 HC	38.8 (11.4)	(a) 72.2 ± 75.0; (b) 2.7 ± 2.0	Yes	Sad, happy, neutral	Subliminal emotion paradigm. Masked Ekman faces. Task: evaluative ratings of the neutral mask face (valence) + offline detection task.	Prime: 33 ms; mask: 467 ms	Whole brain, ROI

Townsend *et al*. 2010	[40]	15 MDD, 15 HC	46.6 (11.2)	(a) 176.4 ± 159.6; (b) 3 (median)	No	Sad, fearful	Emotion face-matching task. Ekman faces. Explicit task: match emotion. Control task: match shapes.		Whole brain, ROIs

Victor *et al*. 2010	[29]	22 MDD (16 MDDr), 25 HC	33.2 (5.0)	Not reported	No	Sad, happy, neutral	Subliminal emotion paradigm. NimStim set of facial expressions. Task: remember the neutral target face and respond to indicate whether this target face appears during the current trial.	Prime: 26 ms; mask: 107 ms	Whole brain, ROI

Zhong *et al*. 2011	[36]	29 MDD, 31 HC, (26 CV subjects)	20.45 (1.82)	Not reported	No	Fearful, angry	Emotion face-matching task. Standardized set of Chinese facial expressions. Implicit task: match faces. Control task: match shapes.	5 s	ROI, Whole brain

Functional connectivity studies:									

Almeida *et al*. 2009	[47]	16 MDD, 16 HC, (15 BDD)	32.3 (9.7)	(a) 13.4 ± 9.6; (b) not reported	Yes	Sad, happy, neutral	Facial expression processing paradigm. Ekman faces. Morphed 50% and 100% intensity. Explicit task: label emotion.	2 s	Dynamic causal modeling

Carballedo *et al*. 2011	[48]	15 MDD, 15 HC	39.87 (8.57)	Not reported	No	Sad, angry	Emotion face-matching task. Ekman faces. Explicit task: match emotion. Control task: match shapes.	5.25 s	Structural equation modeling

Chen *et al*. 2008	[49]	19 MDD, 19 HC	34.3 (8.6)	Not reported	No	Sad	Facial expression processing paradigm. Ekman faces. Morphed to express low, medium and high intensities. Implicit task: indicate the sex of the face.	3 s	Functional connectivity

Dannlowski *et al*. 2009	[50]	34 MDD, 31 HC	38.6 (12.2)	(a) 125.0 ± 125.5; (b) 4.7 ± 5.3	Yes	Sad, angry, happy, neutral	Passive face viewing paradigm. Ekman faces.	500 ms	Functional connectivity

Frodl *et al*. 2010	[51]	25 MDD, 15 HC	39.4 (10.4)	(a) 51.8 ± 63.9; (b) 1.52 ± 0.6	No	Sad, angry	Emotion face-matching task. Ekman faces. Explicit task: match emotion. Implicit task: match gender. Control task: match shapes.		Functional connectivity

Mathews *et al*. 2008	[28]	15 MDD, 16 HC	24.5 (5.5)	(a) not reported; (b) 4.46	No	Angry, fear, happy	Emotion face-matching task. Emotional faces. Task: match faces.	5 s	Functional connectivity

BDD, bipolar disorder; BDDr, bipolar disorder remitted; CV, cognitive vulnerability; MDDr, major depressive disorder remitted; ROI, region of interest

### Neurobiological differences in 'activation' by emotional faces

#### Abnormal limbic activity

##### Amygdala

Of the 20 included fMRI studies, 10 reported significant differences in amygdala responsiveness in MDD patients compared to HCs during exposure to facial emotions. Two recent studies by Victor *et al*. [29] and Suslow *et al*. [30], both using subliminal stimuli presentation, reported a similar differential response pattern of higher amygdala response to sad facial stimuli and decreased responses to happy facial stimuli in MDD patients compared to HCs. Related to negative stimuli, supporting findings were reported earlier by Surguladze *et al*. [31] and Fu *et al*. [32,33]; both groups observed amygdala hyperactivation to overtly presented sad facial expressions. In addition, increased amygdala activation to fearful facial expressions was reported by Sheline *et al*. [34]. The result of amygdala hyper-responsiveness to sad/fearful faces (combined contrast) was again supported by Peluso *et al*. [35] and recently for fearful/angry faces (combined contrast) by Zhong *et al*. [36]. Two results contradicting this pattern should also be mentioned: first, decreased amygdala activation in response to fearful faces in MDD patients compared to HCs in a study investigating bipolar patients as a second control group [37] and second, increased activation to happy facial stimuli [34]. Finally, Matthews *et al*. [28] described in a comparatively young patient sample with early depression onset hyperactivation of the amygdala in MDD patients versus HCs in a combined contrast including fear, angry and happy faces.

In summary, half of the 20 relevant studies report increased amygdala activation in response to emotional faces in MDD patients compared to HCs. Across the aforementioned studies, results indicate predominantly hyper-responsiveness to negative facial expressions, in particular to sadness. Available data on subliminal happy facial processing further suggests hyporesponsiveness of the amygdala in MDD patients.

##### Hippocampus

Although several activations observed in parts of the amygdala extended to (para)hippocampal regions [33,37], only one activation peak has been observed directly in the hippocampus [38]. The observed result showed decreased hippocampus activity to sad facial expressions in MDD patients.

##### Insula, parahippocampal gyrus/thalamus

So far, only one study by Surguladze *et al*. [39] investigated responses to faces displaying different degrees of disgust in MDD patients vs HCs. The authors observed greater activation in the left insula in depressed patients compared to HCs. Apart from altered processing of disgust in MDD patients, additional altered activation to other emotional faces has been reported in the insula: Suslow *et al*. [30] demonstrated higher insula and parahippocampal gyrus (PHG) activation to sad faces and decreased activation to happy faces. This was supported by the results of earlier studies indicating the same direction of insula and PHG responsiveness to sad and happy stimuli, respectively. Zhong *et al*. [36] observed increased insula activation to fearful/angry (combined contrast) faces in a young sample of MDD patients. Additionally, thalamic hyper-responsiveness to sad facial stimuli has been reported by Fu *et al*. [32].

There is a clear trend for similar activation patterns between the insula, PHG area and amygdala, supporting the hypothesis of an emotion bias in limbic structures in MDD patients, with hyper-responsiveness to negative facial expressions and hyporesponsiveness to happy facial expressions. Nevertheless, one group detected decreased activity in the insula in a combined contrast of sad and fear [40] which differs from this pattern.

##### Striatum

Aberrant activity in striatal structures also resembles the activation pattern observed in the amygdala and insula. Again, predominant putamen/caudate nucleus hyper-responsiveness to sad/angry facial expressions and rather hyporesponsiveness in response to happy facial expressions has been observed [32,33,37,41].

#### Abnormal frontal activity

##### Motor cortex and prefrontal cortex

Initially, there is good agreement among the results reported for the motor cortex, a brain area that has been given little attention in emotion processing. Hyperactivated motor cortex (Brodmann's area (BA) 6, BA 4) during sad and angry facial processing in MDD patients compared to HCs was reported by four studies [32,33,41,42]. Findings in the lateral prefrontal cortex (PFC) are less consistent: comparing aberrant increased to decreased activation to sad and angry facial stimuli in DLPFC in MDD patients, we find both reported nearly equally often [30,36,37,42,43]. Similar inconsistencies were reported regarding neural responsiveness to happy facial stimuli in DLPFC and in more ventral, lateral PFC areas (see Table 2 for details). Even though altered neuronal responses in DLPFC are a prevalent finding in MDD patients, it is hardly possible to draw a final conclusion about a general hyper/hypoactivation of the DLPFC during facial emotion processing in unipolar depression, underlining the variability in neuroimaging results. In OFC several independent studies detected decreased activation in inferior and medial OFC areas in response to either sad, fear or angry facial stimuli [37,38,42]. Furthermore, Surguladze *et al*. [39] reported hyperactivation to disgust in OFC in MDD patients.

##### Cingulate gyrus

Aberrant activation in the posterior, mid and anterior cingulum in MDD patients compared to HCs has been almost solely reported to facial expressions of sadness.

**Figure 2:**
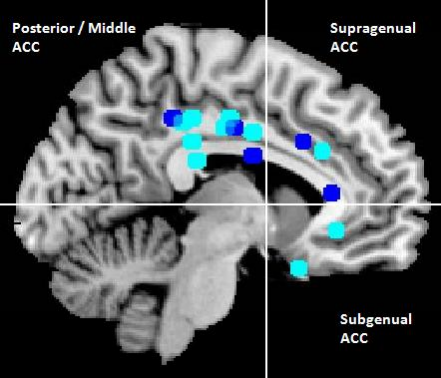
**Increased and decreased cingulate activation in major depressive disorder (MDD) patients in emotional face processing studies**. Paramedian slice of a Montreal Neurological Institute (MNI) template depicting abnormal cingulate activation during emotional face processing in major depressive disorder. Peak activation coordinates reported by primary authors in Talairach coordinates were converted into MNI space. Light blue: hyperactivation, dark blue: hypoactivation. ACC = anterior cingulate cortex.

Findings in posterior cingulate responsiveness are contradictory: Fu *et al*. [32] and Keedwell *et al*. [42] reported enhanced activity in the posterior cingulum, whereas in a later therapy study by Fu and colleagues [33] weakened activity in MDD patients compared to HCs in closely related areas emerged. In the middle cingulate gyrus, two independent studies point to rather enhanced neural responses to sad/angry facial stimuli in MDD patients compared to HCs [32,43]. Of particular concern in the pathophysiology of affective disorders is the role of the ACC [23,44]. Decreased responses to sad facial stimuli in MDD patients compared to HCs in dorsal parts of the ACC were reported by Lawrence *et al*. [37] and Fu *et al*. [33]. However, one study revealed a contradictory finding of rather increased responsiveness in dorsal ACC to sad facial expressions [32]. Interestingly, Gotlib *et al*. [45] reported two hyperactivated clusters in different subgenual parts of the ACC in the MDD group to sad and happy facial expressions, respectively. Figure 2 presents a summary of altered activation loci for facial emotion processing tasks within the posterior, middle and anterior cingulum in unipolar depression.

#### Abnormal temporal activity

##### Lateral: middle temporal gyrus (MTG: BA 21), inferior temporal gyrus (ITG: BA 20), superior temporal gyrus (STG: BA 22, 42)

In MDD patients, several hyperactivations in MTG, ITG and STG in response to sad facial stimuli have been detected [30,33,42], contrary to two observed hypoactivated clusters. In addition, noticeable deactivation to happy facial expressions stimuli has been observed, too [40,45]. Specific to the emotion of disgust, Surguladze *et al*. [39] described increased activation in MTG and STG in MDD patients.

##### Medial: fusiform gyrus/fusiform face area (BA 37)

Suslow *et al*. [30] and Surguladze *et al*. [31] both reported a pattern of increased activation to sad facial expression and decreased activation to happy facial expression in fusiform gyrus in MDD patients compared to HCs. One study supported this pattern [42], also observing increased activation to sad facial stimuli, whereas a deactivation in fusiform gyrus during sad facial processing has also been detected [33].

**Table 2:** Emotional face processing studies: between group fMRI findings (major depressive disorder (MDD) > healthy controls (HCs))

Brain region	BA	Sad > Baseline	Fear > Baseline	Angry > Baseline	Happy > Baseline	Disgust > Baseline	Author/year	Reference
Limbic lobe								

Amygdala		↑					Fu *et al*. 2004	[32]

Amygdala			↑	↑			Peluso *et al*. 2009	[35]

Amygdala			↑^a^		↑^a^		Sheline *et al*. 2001	[34]

Amygdala		↑			↓		Suslow *et al*. 2010	[30]

Amygdala		↑			↓		Victor *et al*. 2010	[29]

Amygdala			↑	↑			Zhong *et al*. 2011	[36]

Extended amygdala			↑	↑	↑		Matthews *et al*. 2008	[28]

Amygdala/hippocampus		↑					Fu *et al*. 2008	[33]

PHG/amygdala		↑^b^					Surguladze *et al*. 2005	[31]

Amygdala/hippocampus			↓				Lawrence *et al*. 2004	[37]

Hippocampus		↓					Lee *et al*. 2008	[38]

Extended limbic system								

Insula		↑					Fu *et al*. 2004	[32]

Insula	13				↓		Gotlib *et al*. 2005	[45]

Insula		↑					Keedwell *et al*. 2005	[42]

Insula						↑	Surguladze *et al*. 2010	[39]

Insula	13		↑	↑			Zhong *et al*. 2011	[36]

Insula		↓	↓				Townsend *et al*. 2010	[40]

Insula/PHG		↑			↓		Suslow *et al*. 2010	[30]

PHG		↑					Fu *et al*. 2008	[33]

PHG/globus pallidus/anterior thalamus			↓		↓		Lawrence *et al*. 2004	[37]

Thalamus		↑					Fu *et al*. 2004	[32]

Striatum								

Putamen		↑					Fu *et al*. 2008	[33]

Putamen		↑^b^			↓^b^		Surguladze *et al*. 2005	[31]

Putamen		↓	↓				Townsend *et al*. 2010	[40]

Putamen/globus pallidus		↑					Fu *et al*. 2004	[32]

Uncus/amygdala/caudate/putamen					↓		Lawrence *et al*. 2004	[37]

Caudate		↑					Fu *et al*. 2004	[32]

Caudate		↓					Lee *et al*. 2008	[38]

Caudate		↑		↑			Scheuerecker *et al*. 2010	[41]

Frontal lobe								

Motor cortex								

Premotor cortex	6	↑					Fu *et al*. 2004	[32]

Middle frontal gyrus	6	↑					Fu *et al*. 2008	[33]

SMA		↑		↑			Scheuerecker *et al*. 2010	[41]

Precentral gyrus	4	↑					Fu *et al*. 2004	[32]

Precentral gyrus	4	↑					Fu *et al*. 2008	[33]

Precentral gyrus	4				↑		Keedwell *et al*. 2005	[42]

Precentral gyrus	4,6				↓		Keedwell *et al*. 2005	[42]

Precentral gyrus		↑		↑			Scheuerecker *et al*. 2010	[41]

Postcentral gyrus	1, 2, 3	↑					Fu *et al*. 2004	[32]

Postcentral gyrus		↑		↑			Frodl *et al*. 2009	[43]

Postcentral gyrus	2	↑					Keedwell *et al*. 2005	[42]

DLPFC								

DLPFC	44, 45, 9	↓	↓		↓		Lawrence *et al*. 2004	[37]

DLPFC	9	↑					Keedwell *et al*. 2005	[42]

Superior frontal gyrus		↑^a^		↑			Frodl *et al*. 2009	[43]

Superior frontal gyrus	8				↑		Gotlib *et al*. 2005	[45]

Superior frontal gyrus	8	↓					Fu *et al*. 2008	[33]

Superior frontal gyrus	8		↓	↓			Zhong *et al*. 2011	[36]

Middle frontal gyrus		↑^a^		↑			Frodl *et al*. 2009	[43]

Middle frontal gyrus		↑			↓		Suslow *et al*. 2010	[30]

Middle frontal gyrus	8	↓					Fu *et al*. 2008	[33]

Middle frontal gyrus	8	↓					Keedwell *et al*. 2005	[42]

VLPFC								

VLPFC	10, 47, 45, 46				↓		Lawrence *et al*. 2004	[37]

VLPFC	11				↑		Gotlib *et al*. 2005	[45]

VLPFC	10/47	↓					Keedwell *et al*. 2005	[42]

Middle frontal gyrus	10/47				↑		Keedwell *et al*. 2005	[42]

Middle frontal gyrus		↑		↑			Scheuerecker *et al*. 2010	[41]

Cingulum								

Anterior cingulum	32	↑					Fu *et al*. 2004	[32]

Anterior cingulum	24/32	↓					Fu *et al*. 2008	[33]

Anterior cingulum	25		↓	↓			Zhong *et al*. 2011	[36]

Sg anterior cingulum	25	↑					Gotlib *et al*. 2005	[45]

Sg anterior cingulum	24/32				↑^a^		Gotlib *et al*. 2005	[45]

Anterior cingulum	24	↓					Lawrence *et al*. 2004	[37]

Middle cingulum	23/24	↑					Fu *et al*. 2004	[32]

Middle cingulum	33/24, 32/24	↓					Fu *et al*. 2008	[33]

Middle cingulum		↑^a^		↑			Frodl *et al*. 2009	[43]

Middle cingulum	23				↑		Keedwell *et al*. 2005	[42]

Posterior cingulum	23/31, 29/31	↑					Fu *et al*. 2004	[32]

Posterior cingulum	31	↓					Fu *et al*. 2008	[33]

Posterior cingulum	31				↓		Fu *et al*. 2007	[63]

Posterior cingulum	31	↑					Keedwell *et al*. 2005	[42]

Medial PFC								

Inferior frontal gyrus	47	↑					Gotlib *et al*. 2005	[45]

Inferior frontal gyrus	47/45	↓					Gotlib *et al*. 2005	[45]

Inferior frontal gyrus	45	↑					Keedwell *et al*. 2005	[42]

Inferior frontal gyrus	47		↓	↓			Zhong *et al*. 2011	[36]

Medial PFC	10, 11, 47		↓				Lawrence *et al*. 2004	[37]

DMPFC	8				↑		Keedwell *et al*. 2005	[42]

VMPFC	10/32				↑		Keedwell *et al*. 2005	[42]

Orbitofrontal cortex								

Orbitofrontal cortex	47					↑	Surguladze *et al*. 2010	[39]

Orbitofrontal cortex	11	↓					Lawrence *et al*. 2004	[37]

Orbitofrontal cortex	11	↓					Keedwell *et al*. 2005	[42]

Orbitofrontal cortex		↓		↓			Lee *et al*. 2008	[38]

Orbitofrontal cortex		↓		↓			Lee *et al*. 2008	[38]

Temporal lobe								

Middle temporal gyrus	21	↓					Gotlib *et al*. 2005	[45]

Middle temporal gyrus	21/22				↓		Keedwell *et al*. 2005	[42]

Middle temporal gyrus	21					↑	Surguladze *et al*. 2010	[39]

Middle temporal gyrus		↑			↓		Suslow *et al*. 2010	[30]

Middle temporal gyrus/inferior temporal gyrus		↑			↓		Suslow *et al*. 2010	[30]

Middle temporal gyrus	21, 37	↓					Townsend *et al*. 2010	[40]

Inferior temporal gyrus	20				↓		Gotlib *et al*. 2005	[45]

Inferior temporal gyrus	37					↑	Surguladze *et al*. 2010	[39]

Inferior temporal gyrus	20	↓					Townsend *et al*. 2010	[40]

Superior temporal gyrus	42	↑					Fu *et al*. 2008	[33]

Superior temporal gyrus	42	↑					Keedwell *et al*. 2005	[42]

Fusiform gyrus	37	↓					Fu *et al*. 2008	[33]

Fusiform gyrus	20	↑					Keedwell *et al*. 2005	[42]

Fusiform gyrus	19	↑^b^			↓^b^		Surguladze *et al*. 2005	[31]

Fusiform gyrus		↑			↓		Suslow *et al*. 2010	[30]

The table includes whole brain and region of interest (ROI) results of the described studies in Table 1, section 'Whole Brain and ROI studies'. The table does not include results on other study aspects such as treatment response or connectivity analyses.

^a^Due to deactivations in controls.

^b^Linear trend for increasing intensities of sadness/happiness.

BA, Brodmann's area; DLPFC, dorsolateral prefrontal cortex; DMPFC, dorsomedial prefrontal cortex; PFC, prefrontal cortex; PHG, parahippocampal gyrus; SMA, supplementary motor area; Sg, subgenual; VLPFC, ventrolateral prefrontal cortex; VMPFC, ventromedial prefrontal cortex.

### Differential effects of valence (positive versus negative facial emotions)

In limbic regions, the combined results of aberrant negative face processing in MDD patients revealed predominantly exaggerated responsiveness of the amygdala, PHG and insula (for details, see section above). In striatal regions, further increased responsiveness to negative stimuli has been detected in putamen and caudate nucleus [31-33,41]. By contrast, data on the processing of positive facial stimuli rather indicate decreased responsiveness in MDD patients compared to HCs in the amygdala, insula, PHG and putamen [29-31,37,45]. In frontal lobe structures a deviant neural response picture emerged: exaggerated responses to negative facial stimuli in MDD patients occurred particularly in the motor cortex [32,33,41,42] and in the middle and subgenual cingulum (see Figure 2), whereas rather decreased responsiveness was dominant in the OFC [37,38,42]. Concerning the processing of positive facial stimuli in frontal areas, increased as well as decreased activity has been observed in MDD patients (see Table 2). Thus, the present data suggest group × valence interactions particularly in areas involved in the generation of affective responses, indicating a neurobiological substrate of mood-congruent processing bias. However, unfortunately only a few studies explicitly investigated group × valence interactions in factorial designs. Brain areas showing group × valence interactions include the amygdala [29,30], insula, PHG [30], the fusiform gyrus [30,31] and putamen [31].

### Connectivity results

Connectivity analysis in functional neuroimaging can be subdivided into two general classes: functional connectivity (FC), which examines simple correlations between neural activity in two anatomically distinct brain areas; and effective connectivity (EC), which measures the directional influence that one neural system exerts over another [46]. The most influential models of depressive disorders assume that depressive symptoms might rather result from abnormal interactions between several brain regions than from differences in single (isolated) local brain function [7,23,25]. However, relatively few functional neuroimaging studies have investigated connectivity within these postulated networks. To date, six studies have examined neural connectivity in MDD patients compared to HCs during the processing of facial expressions [28,47-51]. One of the first studies by Chen *et al*. [49] in 19 unmedicated patients with MDD and 19 HCs used regression analysis between the amygdala and all other brain regions on neural activity to sad facial expressions. The authors found decreased FC of bilateral amygdala in depressed patients compared to matched HCs in the hippocampus, putamen, insula, PHG, inferior, middle, and superior temporal cortices, and inferior/middle frontal cortices before antidepressant treatment. Antidepressant treatment was associated with a significant increase in FC between the amygdala and right frontal cortex, supragenual ACC, striatum and thalamus in MDD subjects. Matthews *et al*. [28] focused explicitly on differences in amygdala-cingulate FC during emotional face processing in 15 MDD patients and 16 HCs. The results indicate increased FC between the bilateral amygdala and subgenual ACC and decreased FC between the amygdala and supragenual ACC in MDD patients. Furthermore, greater depressive symptom severity was positively correlated with decreased coactivation of the supragenual cingulate in MDD subjects. Dannlowski *et al*. [50] calculated FC of the amygdala with prefrontal areas based on neural activity during passive viewing of negative emotional faces in a large sample of 34 MDD patients with relatively long illness history and 32 HC subjects. Depressed patients showed significantly reduced connectivity of the amygdala with the dorsal ACC and DLPFC. Taken together, all three FC studies show comparable results concerning abnormally reduced FC between the amygdala and dorsal/supragenual ACC regions in acute depression, while amygdala-subgenual ACC connectivity seems to be increased. Recently, Frodl *et al*. [51] selected the OFC instead of the amygdala as the 'seed region' for functional connectivity analysis. In 15 unmedicated MDD patients and 15 HC subjects, functional connectivity between the OFC and other brain regions was assessed during negative facial emotion processing. Results between patients and HCs demonstrate that the OFC coactivated less with the dorsal ACC, precuneus, and cerebellum and more with the right DLPFC, right inferior frontal operculum, and left motor areas in the patient group.

To the best of our knowledge, facial emotion processing in MDD patients has only twice been investigated with effective connectivity techniques. Almeida *et al*. [47] used dynamic causal modeling (DCM) to examine EC between the amygdala and medial OFC. Their data showed reduced left-sided top-down OFC-amygdala EC in the happy and sad facial processing paradigm in a sample of predominantly female depressed subjects compared to HCs. Recently, Carballedo *et al*. [48] used structural equation modeling (SEM) to calculate the differences in effective connectivity between 15 MDD patients and 15 HCs. The authors proposed an emotional model including the amygdala, OFC, ACC and PFC. Bilaterally, the path analysis revealed attenuated connectivity strengths from the amygdala to OFC during sad and angry facial processing in patients compared to controls. Additionally, for the right hemisphere, patients show lower connectivity from the amygdala to the ACC and from ACC to PFC, whereas controls show lower connectivity in the opposite direction, namely from ACC to the amygdala. One should note that both EC studies reviewed here found lower left-sided influences between the amygdala and OFC, although the study by Almeida *et al*. [47] showed top-down alterations and the one by Carballedo *et al*. [48] bottom-up alterations.

In summary, functional connectivity between the amygdala and other brain areas shows (a) decreased amygdala coupling with other limbic regions (hippocampus, putamen, insula, PHG), temporal regions, and in particular with the supragenual/dorsal ACC and DLPFC, and (b) increased coupling with subgenual ACC. Of particular concern seems to be the role of the ACC, resembling results identified with conventional fMRI analysis. The longitudinal data by Chen *et al*. [49] provide first evidence that decreased FC coupling between the amygdala and supragenual ACC increases after pharmacological intervention.

## Discussion

The present review aimed to summarize available empirical data regarding the neural correlates of abnormal emotional face processing in acute unipolar depression (during the current episode). Presenting differential facial expressions activates a common face-processing network in HCs and MDD patients, including primary visual pathways as well as further supporting brain areas crucial for emotion processing in general. The amygdala belongs to the latter group, the extended limbic system and specific frontal areas, namely the ACC, OFC and ventromedial prefrontal cortex (VMPFC). These regions are of particular interest for understanding the pathophysiology of unipolar depression. Our analysis indicates evidence of abnormal neural face processing in MDD patients, especially in the amygdala, the insula, PHG, ACC and OFC. Although neural alterations were reported in several other brain regions, the Discussion section focuses on these areas because they are (a) crucial for evaluating the neural mood-congruent face processing hypothesis, and (b) are core domains in an altered functional connectivity network in MDD patients during emotional face processing.

### Neural mood-congruent face processing

Neural responses in MDD patients associated with mood-congruent processing patterns are most evident in the amygdala [29,30], insula and PHG [30], the fusiform gyrus [30,31] and putamen [31].

The amygdala plays a pivotal role in emotion processing and in the perception and processing of emotional salience in facial expressions (for reviews see [52-54]). Furthermore, the amygdala is a key region within the neurobiological framework of depressive disorder. Several authors have suggested that, for MDD, mood-congruent bias in behavioral measures is strongly linked to amygdala hyper-responsiveness to negative stimuli [2,55,56]. Findings of increased amygdala responsiveness to negative emotional faces are well in line with several imaging studies employing other stimuli, including negative words [57,58], individualized self-referential sentences [59], or in expectation of negative pictures [60]. Furthermore, these findings are supported by studies in depressed adolescents [61].

However, not all fMRI studies have found evidence for altered amygdala activation in MDD. In detail, 10 of the 20 included studies reported differences in amygdala activation between MDD patients and HCs using face emotion processing tasks [28-37], while the other studies found no significant group effects [27,38-43,45,62,63]. Nevertheless, focusing on the observed differences in amygdala responsiveness, studies provide compelling support for amygdala mood-congruent processing in MDD patients. First, abnormal amygdala responsiveness has been shown to negative and positive facial expressions, corroborating amygdala function in processing salient stimuli, independent of stimuli valence [64]. Second, as hypothesized in mood congruent processing theories of depression, the majority of results show exaggerated amygdala response to sad stimuli, and in addition decreased amygdala response to happy facial stimuli, although replications with happy facial expressions are still rare. These results indicate that, in convergence with behavioral measures, neurobiological assessment can be a sensitive measure for mood-congruent biases in unipolar depression. Of note are two recent studies using subliminal presentation conditions pointing to mood congruency effects to negative and positive stimuli already at early, automatic processing stages [29,30].

In conclusion, the findings of our analysis support the assumption that amygdala hyperactivity is associated with negatively biased facial emotion processing implicated in the pathophysiology of major depression, although this became evident in only one-half of the reviewed studies. Studies investigating the question of whether abnormalities in amygdala responses to emotional faces demonstrated in acute depression represent a state marker of acute depressive episodes or vulnerability factors for depression are rare. In remitted patients, Neumeister *et al*. [65] demonstrated enhanced regional cerebral blood flow responses to sad facial expressions in the amygdala relative to HCs, but others have failed to replicate these findings in remitted patients [66,67]. In people at risk for depression, van der Veen *et al*. [68] and Monk *et al*. [69] reported greater abnormal amygdala activation to negative facial expression, but again inconsistent findings exist [70]. Interestingly, Zhong *et al*. [36] reported higher amygdala activation evident in both MDD subjects and a sample of healthy people with high cognitive vulnerability to depression compared to HCs. Increased left amygdala responsiveness was positively associated with CSQ scores (measures causal attributions, consequences and self-worth characteristics). In addition, Cremers *et al*. [71] reported that right amygdala-dorsomedial PFC connectivity for negative faces vs neutral faces was positively associated with neuroticism scores, a personality trait related to the development of affective disorders. Finally, a recent study by Dannlowski *et al*. [72] investigated long-term effects of childhood maltreatment with fMRI in psychologically healthy participants. The observed association between childhood maltreatment and amygdala responsiveness during emotional face processing resembles findings in depressed patients, suggesting that these functional changes might constitute a predisposition for developing affective disorders.

Hyperactivated amygdala to negative emotional faces in remitted patients and people at high risk for depression is indicative of trait vulnerability. This interpretation receives support from imaging genetics and twin studies, suggesting that amygdala responsiveness to emotional faces as well as amygdala prefrontal connectivity are under strong genetic influence [73-78].

Some methodological aspects explaining the heterogeneity of studies should be discussed here. With regard to presentation modus, all three studies using subliminal presentation of facial expressions reported differences in amygdala activation [29,30,34]. Victor *et al*. [29] even observed differences in amygdala activation specific to masked presentation of sad and happy faces, absent to unmasked stimuli, supporting the assumption that subliminal stimuli presentation maybe an advantage in identifying emotional-processing biases in MDD with focus on amygdala activation. It may be subliminal stimulus presentation prevents confounding with other cognitive processes prevalent in depression such as rumination on negative thoughts/preservation of attention to negative faces [34]. Comparing paradigms presenting facial stimuli supraliminally, only about half of the investigations implementing either face-matching paradigms or the 'face recognition task' observed amygdala differences. Scheuerecker *et al*. [41] suggested that participants probably used more visual and cognitive strategies to solve the face-matching task, causing ACC and PFC activation maybe inhibiting amygdala activation. Concerning task type (that is, explicit or implicit), an implicit task, requiring participants to focus on gender aspects of the face, seems to be sufficient to elicit amygdala activation [28,31-33,35,36]. As amygdala and frontal responsiveness depends on task complexity, face type and attention focus, future research should take into account such variations in designing facial processing paradigms.

Furthermore, medication status has an important impact on neural activation patterns: seven of the ten studies reporting altered amygdala activation were performed on unmedicated patients. This result is not surprising regarding the converging evidence, that amygdala is a key region for antidepressant effects, reducing abnormal amygdala responsiveness to negatively valenced faces in MDD patients (for a recent meta-analysis see [79]). Other possible influencing factors may be methodological aspects such as experimental design (for example, event-related vs block design) or the selection of different baseline conditions (for example, neutral faces or a no-face condition) as well as clinical and non-diagnostic variables such as age, comorbidity, treatment history and number of prior episodes (for details see Table 1). Furthermore, difficulties in detecting altered amygdala responsiveness in MDD patients may be caused by a 'ceiling' effect. As noted by Townsend *et al*. [40], several PET studies have shown increased resting blood flow in the amygdala in MDD patients [80-84], making it difficult to detect group differences in activation tasks if amygdala baseline activation was already increased.

Aside from the amygdala, several other subcortical brain structures show activation patterns supporting mood-congruent processing in depressed patients. Insula hyperactivation to sad facial stimuli is a prominent result, and furthermore two independent studies observed hypoactivation to happy facial stimuli (see Table 2). Apart from having a pivotal role in the processing of disgust [39] the insula has strong functional connections to the amygdala [85]. Insula projections to inferior parietal cortex and the amygdala are involved in identifying/representing motivational salient information, social cues and the expression of conditioned responses: particularly on implicit processing pathways [86,87]. Furthermore, activity in the putamen and caudate nucleus also resembles mood-congruent activation patterns in MDD patients, although contributions to the processing of facial expressions are still under debate [87]. In visual face areas, fusiform gyrus responsiveness also indicates mood-congruent processing in terms of increased activation to sad facial expressions and decreased activation to happy faces. In addition to encoding face traits and facial identity [20], recent studies revealed that fusiform regions are also sensitive to facial emotional expression (for a review see [88]). The authors suggest that the modulation by emotional effects can be explained by direct connections between visual cortex and the amygdala, facilitating direct feedback signals from the amygdala [89] to visual processing areas.

In summary, neuronal correlates of mood-congruent facial affect processing in MDD patients are most prominent in limbic and subcortical regions, compromising the amygdala, insula and putamen/caudate nucleus. In a larger context these regions are hypothesized to be part of an extended emotional face processing system [20] and furthermore constitute a ventral stream in emotion-cognition processing, appraising emotional behavior and producing affective states, altered in unipolar depression [25]. As described, these alterations may even influence visual processing areas such as the fusiform face gyrus. Studies in remitted patients and in people at risk for depression provide the first indications that enhanced limbic neural responses to negative emotional material may contribute to vulnerability to MDD [65,68,69].

### Abnormal ACC and OFC activity

**Figure 3:**
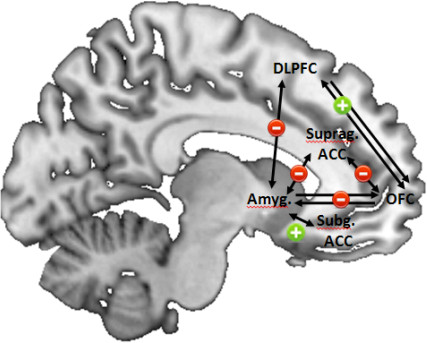
**Schematic illustration of main results reported in fMRI connectivity studies on aberrant emotional face processing in major depressive disorder (MDD) patients**. Double arrows represent results derived by functional connectivity approaches, whereas the normal arrows present the result derived by effective connectivity analyses. Plus and minus characters indicate increased and decreased connectivity between brain regions in MDD. ACC = anterior cingulate cortex; Amyg = amygdala; DLPFC = dorsolateral prefrontal cortex; OFC = orbitofrontal cortex; suprag = supragenual; subg = subgenual.

The analysis of whole-brain and functional connectivity data highlight two more regions showing abnormal activation patterns during emotional face processing in MDD: the cingulate gyrus and the orbitofrontal cortex. Findings in the cingulate gyrus derived by our whole-brain and ROI analysis (see Figure 2) can be broadly subsumed by hyperactivated posterior/middle cingulum, hypoactivated dorsal anterior cingulum and hyperactivated ventral/subgenual anterior cingulum in MDD patients compared to HCs, although findings are less clear for different subregions of the ACC than expected. Several authors postulate a central role particularly for the ACC in the neurobiology of depression, with a special role in therapy response [90-92]. The ACC plays a crucial role for attentional processes that integrate cognitive and emotional processes. While the subgenual ACC seems to be involved in the generation and recognition of emotional states, the supragenual/dorsal ACC seems to be crucial for emotion regulation [25,93,94]. Functional connectivity results between amygdala and subgenual/supragenual ACC on emotional face processing extend the above described neural activation pattern: while (hypoactivated) dorsal regions of the ACC show decreased FC with the amygdala, the rather hyperactivated subgenual parts seem to have increased connectivity with the amygdala ([28,49,50]; see Figure 3). On the one hand, cognitive parts of the ACC are less activated in MDD patients compared to HCs during emotional face processing and show decreased FC to the amygdala, suggesting less capability in MDD patients to modify/suppress emotional salient information crucial for patients' affective states and behavioral responses. On the other hand, connections between subgenual parts of the ACC and the amygdala are increased, maybe mutually enhancing abnormal emotion processing. Future studies should address the direction of influence between different parts of the ACC and the amygdala in more detail, preferably using EC methods and more refined models. A recent example is the EC study by Carballedo *et al*. [48], pointing to lower connectivity strength from the amygdala to the ACC in patients.

Moreover, results on medication effects concerning abnormal ACC activity should also be taken into account and can extend the interpretations. Antidepressant medication reduced ACC activity in HCs during an emotion provoking paradigm [95] and Pizzagalli recently highlighted in his meta-analysis that increased rostral ACC activity at rest is a strong marker of treatment response in depression [96]. Because these data are mainly derived from acutely depressed patients, it is still unknown whether abnormal ACC responses represent state markers of depression or a vulnerability factor [96]. The first evidence supporting the latter notion comes from the previously mentioned study by Cremers *et al*. [71]. The authors reported a negative correlation between amygdala-ACC connectivity and neuroticism to negative faces compared to neutral faces, possibly indicating that highly neurotic patients are characterized by less inhibitory control of the ACC over the amygdala, which may reflect vulnerability for MDD. A second study in young people at risk for depression would support a possible diminished cortical regulation of negative emotional faces [70].

With respect to the OFC, whole-brain and ROI analyses display remarkably decreased neural activity in MDD patients compared to HCs in medial OFC areas to negative facial stimuli [37,38,42]. In addition, FC between the OFC and other brain areas revealed decreased FC to the amygdala and supragenual ACC as well as increased FC to the DLPFC. The two available EC studies [47,48] in MDD patients further specified the directionality of these brain abnormalities. Both studies indicate reduced left-sided connectivity between the OFC and the amygdala in patients, but show, at first glance, contradictory results with regard to the direction of influence on another (top down vs bottom up). In both paradigms participants were instructed to explicitly label emotions, but different paradigms were used (face-matching task vs morphed facial expression processing paradigm); therefore this may be, next to medication effects, one reason explaining the results. Future studies are needed to further investigate on this issue.

The OFC is a central part of the frontosubcortical circuits, connecting the frontal and limbic systems with each other, and is crucial for mood regulatory processes [97,98]. Relative uncoupling of connections between heightened activity in the limbic system and the OFC during negative facial processing in MDD may account for depressive symptoms such as negative emotional experiences and impaired regulation of emotional and social behavior [41]. Increased FC between OFC and lateral PFC systems could be the neural substrate of a more voluntary compensatory mechanism in MDD [99] for the described altered automatic emotional face processing.

### Unresolved questions

To date, it is not clear whether the neurobiological abnormalities described above represent state or trait markers of depression. As highlighted above, a few studies have demonstrated a normalization of abnormal neurobiological response patterns after antidepressant medication (for example, [29,32]). Moreover, these studies are in line with several pharmaco-fMRI studies in healthy subjects, showing that limbic responsiveness to negative facial stimuli can be attenuated even by short-term antidepressant administration [100-102]. However, although it seems that antidepressants modify pathological emotional face processing in depression, it still remains to be clarified whether these functional abnormalities in emotional face processing represent a feature of acute depressed state and would therefore also resolve without medication after remission or whether they represent a risk factor preceding the onset of depression. The first studies in remitted patients and in high-risk subjects [36,65,68,69,71,103], as well as data from imaging genetics and twin studies [73-78] suggest that amygdala responsiveness to emotional faces as well as amygdala-prefrontal and amygdala-ACC connectivity may represent vulnerability factors for MDD.

A second unresolved question concerns possible laterality effects of valence-specific emotion processing in the depressed brain. Although this aspect may be raised by the data, it was not the focus of our analysis and still needs further clarification. As noted above, other unresolved issues concern the heterogeneity of presentation paradigms. For example, studies investigating automatic facial emotion processing are likely to target other brain areas compared to explicit emotion processing paradigms. Obviously, this is particularly important for investigating prefrontal areas and might explain the apparently contradictive results in brain areas involved in emotion regulation, for example the DLPFC. Next to the methodological aspect, variability between patient samples due to different symptom characteristics may be a further critical, influential factor. Age, comorbidity, treatment history, number of prior episodes or age on illness onset may confound the reported results [7]. Unfortunately, information about clinical variables was provided by less than half of the reviewed studies, leaving these variables relatively uncontrolled for in this review and therefore limiting the described results and their interpretation. As described in the Discussion section, differences in medication status and low sample sizes could further contribute to inconsistencies among study results.

The research field would benefit from larger studies with well characterized patient samples (that is, detailed description of clinical variables), particularly multicenter studies. Furthermore, investigators should carry on employing standardized paradigms in order to replicate results and to resolve conflicting findings. For example, the comparison of subliminal and supraliminal stimulus presentation in one patient sample and the influence of attentional mechanisms on a neural level are still rarely investigated. Future studies should explicitly focus on group × valence interactions in factorial designs to explore differential effects of valence and should use connectivity analysis strategies (FC and/or EC) to describe the interplay of core regions such as the amygdala, ACC and OFC more precisely. Longitudinal studies, including relatives or other high-risk subjects are very essential and may ultimately answer the question if the described anomalies represent 'trait' or 'state' marker of depression.

Finally, one should notice that facial processing is only one aspect of altered cognitive/emotional processing among several others in MDD described by behavioral (for review see [104]) and neuroimaging (for review see [7]) studies. Thus, one must caution against overinterpretation of the presented results on altered neural facial processing in MDD.

## Conclusions

Based on cognitive models of depression and behavioral studies pointing to an emotion processing bias in acute depression, several neuroimaging studies have investigated the neuronal underpinnings of these emotional processing abnormalities. It has been shown that the use of emotional face processing tasks is a reliable and valid approach to pinpoint most if not all relevant areas. The analysis of neural activation data shows that MDD patients are characterized by abnormalities within the common face processing network, indicating a mood-congruent processing bias particularly in the amygdala, insula and PHG, fusiform face area and putamen responsiveness. Furthermore, abnormalities in the cingulate gyrus and OFC are obvious, which are refined by investigations implementing functional connectivity analysis. A pathologically altered emotion processing and emotion regulation network emerged, including the amygdala, the ACC, OFC and DLPFC as core components. Further neuroimaging studies will be needed to extend these findings, especially by replicating data with same activation paradigms and larger sample sizes in order to enable researchers to make more valid assumptions on neural emotional processing mechanisms, contributing to a better understanding of depressive disorders.

## Competing interests

The authors declare that they have no competing interests.

## Authors' contributions

AS performed the literature research and wrote major parts of the article. TS contributed to the Introduction and Discussion sections. UD selected topics, article structure, and inclusion criteria, supervised the literature research, and wrote major parts of the discussion section. All authors read and approved the final manuscript.
